# Assessing Critical Thinking in Young Children: Development and Validation of the Critical Thinking Assessment for Children

**DOI:** 10.5964/ejop.17833

**Published:** 2026-05-29

**Authors:** Rosa Angela Fabio, Martina Patafi

**Affiliations:** 1 Department of Biomedical Sciences, Dental Sciences and Morpho-functional, University of Messina, Messina, Italy; 2 Department of Clinical and Experimental Medicine, University of Messina, Messina, Italy; University of South Wales, Cardiff, United Kingdom

**Keywords:** critical thinking, child assessment, cognitive development, psychometric validation

## Abstract

In today’s media-saturated environment, young children are increasingly exposed to diverse and sometimes conflicting information. Developing critical thinking skills early is essential for evaluating, interpreting, and making informed judgments. To address this need, we developed and validated the Critical Thinking Assessment for Children (CTAC), an instrument designed to systematically measure critical thinking abilities in primary school learners.

Given the multidimensional nature of critical thinking, item generation followed a rigorous process involving expert evaluation and pilot testing. An initial pilot study with 72 Italian children aged 6 to 8 years led to a refined 22-item version, selected based on item discrimination indices. Study 1 examined the psychometric properties of the CTAC in a sample of 112 children (*M* = 7.43, *SD* = 1.12) across different grades. Results from an exploratory factor analysis identified a three-factor structure: Open-Minded Inquiry, Media Literacy and Critical Analysis, and Contradiction Recognition and Critical Awareness. Study 2 validated this structure in a larger sample of 356 children (*M* = 8.45, *SD* = 1.20), incorporating concurrent validity measures, including Raven’s Coloured Progressive Matrices and teacher-reported academic performance.

Results from a confirmatory factor analysis supported the robustness of a second-order three-factor model. The findings indicate that the CTAC is a reliable and valid tool for assessing critical thinking within the Italian primary school context. Its application can provide educators and researchers with insights into early cognitive development and serve as a foundation for designing targeted educational interventions. Future studies should examine its longitudinal sensitivity and evaluate its adaptability across different cultural and linguistic contexts.

## The Role of Critical Thinking in Cognitive Development

Critical thinking is an active and systematic process of conceptualizing, applying, analyzing, synthesizing, and evaluating information to guide beliefs and actions (Elder & Paul, 2004). It involves the ability to think clearly and rationally, recognize logical connections between ideas, and critically assess information rather than passively accepting it (Mulnix & Mulnix, 2010; Paul & Elder, 2019, 2020). In today’s complex and polarized society, critical thinking serves as a fundamental intellectual tool for distinguishing weak arguments from sound reasoning (Alarcón-López et al., 2024; Nye & Drasgow, 2011). It promotes an open-minded approach free from stereotypes and biases, facilitating the differentiation of well-founded facts from unfounded assumptions and encouraging multiple perspectives (Davies & Stevens, 2019).

Furthermore, critical thinking entails the ability to counter arguments, which may sometimes be misinterpreted as opposition, potentially leading to communication breakdowns and defensive reactions (Alarcón-López et al., 2024; Baron, 2009; Kahneman, 2011; Stanovich, 2011). The increasing information overload due to excessive media exposure further underscores the necessity of filtering content critically (Halpern, 2013).

Given its significance, critical thinking is one of the most studied cognitive abilities in education (Kuhn, 1999; Peter, 2012; Prokop-Dorner et al., 2024; Snyder & Snyder, 2008). Even before formal schooling, children receive vast amounts of information from their environment and social interactions, necessitating the early development of evaluative skills to prevent misinformation (Brosseau-Liard, 2017). Research on informant reliability has shown that children are sensitive to the trustworthiness and expertise of sources, learning to discern reliable from unreliable informants from an early age (Harris, 2012; Koenig & Harris, 2005). Recent studies extend this work to non-human agents, such as robots or virtual assistants, revealing that children also evaluate the credibility of artificial informants and adapt their reliance on information accordingly (Belpaeme et al., 2018; Danovitch & Alzahabi, 2020). Integrating these findings emphasizes the importance of developing evaluative and critical thinking skills in contexts where information comes from diverse, and sometimes non-human, sources. According to Piaget’s cognitive development theory, children aged 2 to 7 years are in the preoperational stage, where thinking is egocentric, intuitive, and symbolic rather than logical, limiting their capacity for critical reasoning (Piaget, 1962). Their cognitive processes are perception-driven, making it challenging to assess multiple perspectives or engage in deeper analysis. During the concrete operational stage (ages 7 to 11), children develop logical reasoning, enabling more sophisticated critical thinking as they decenter and consider others’ viewpoints (Piaget, 1962; Sun & Hui, 2012). This cognitive shift facilitates a more structured approach to evaluating information and making reasoned judgments. In the formal operational stage (ages 12 and above), adolescents acquire the ability to think abstractly, manipulate mental concepts, test hypotheses, and draw conclusions based on reasoning, marking a significant advancement in critical thinking skills (Daniel & Gagnon, 2012).

Expanding on Piaget’s framework, Daniel and Gagnon (2012) proposed the developmental model of dialogical critical thinking for children aged 4 to 12 years, emphasizing the role of dialogue and interaction in fostering logical, creative, responsible, and metacognitive thinking. Their model delineates a progression through six epistemological stages, from egocentricity to intersubjectivity, highlighting the growing sophistication of children’s cognitive processes. Initially, children’s thinking is self-centered and based on subjective experiences; however, with maturation, they incorporate broader social and moral considerations, ultimately reaching a stage where they can critically evaluate and transform perspectives through dialogue (Daniel & Gagnon, 2012). These models collectively underscore the gradual transition from egocentric to evaluative and dialogical thinking, demonstrating the importance of social interaction in cognitive development.

## Challenges in Assessing Critical Thinking in Children

Assessing critical thinking in children presents significant challenges due to the complexity of the construct and the limitations of existing measurement tools. One key issue is the dependency of assessment results on test formats. Multiple-choice tests, although easy to administer and score, may not capture the depth of critical thinking processes (Hyytinen et al., 2015; Ku, 2009). Constructed-response tasks provide richer insights into cognitive processes but are time-consuming and resource-intensive (Ku, 2009).

Performance-based assessments have gained attention due to their real-world applicability. Shavelson et al. (2019) argue that performance tasks offer a more accurate measure of critical thinking by engaging students in authentic problem-solving contexts. Uglanova et al. (2023) developed the Test of Problem Solving 3-Elementary (TOPS 3-E), which evaluates critical thinking through verbal constructed-response tasks in real-life scenarios, although its context-based nature may influence how critical thinking manifests. Similarly, Gelerstein et al. (2016) created a language arts test aligned with the Delphi Report’s theoretical framework, but its reliance on prior subject knowledge limits its generalizability. Fabio et al. (2023) employed performance-based tests involving social dilemmas and mathematical problems, balancing observable variables with assessment duration; however, the limited number of scenarios used may restrict the scope of competencies assessed.

Other instruments, such as the Critical Thinking Disposition Inventory (CTDI; Merma-Molina et al., 2022), measure critical thinking dispositions across six dimensions but rely on self-reported data, raising concerns about reliability. The Holistic Critical Thinking Scoring Rubric (HCTSR; Facione & Facione, 1996, 2013) provides structured criteria for evaluating critical thinking in verbal or written tasks but faces psychometric validity challenges and depends on rater expertise (Crisafulli et al., 2017). The Cornell Critical Thinking Tests (CCTT) Level X, designed for a broad age range, has also been scrutinized for construct validity (Leach et al., 2020). In summary, the assessment of critical thinking in children requires improvements in construct representation, reliability, and validity. While performance-based tools and rubrics show promise, they each present specific limitations that must be addressed to enhance their applicability.

## Hypotheses

The general aim of this study is to develop a novel measurement tool for assessing critical thinking in children, addressing the limitations of existing instruments. Drawing on established theoretical frameworks and evaluating the tool’s psychometric properties, we propose the following hypotheses:

*Hypothesis 1:* It is hypothesized that the newly developed tool (CTAC) will demonstrate strong psychometric properties, including satisfactory internal consistency, factorial validity, and convergent validity with external cognitive measures. The tool is expected to provide a more comprehensive representation of critical thinking in children than what is typically achieved with existing instruments.
*Hypothesis 2:* We propose that the tool will be able to distinguish developmental differences in critical thinking abilities across age groups, in line with Piaget’s (1962) developmental stages and Daniel and Gagnon’s (2012) theories. Younger children (ages 6–7) are expected to show less advanced critical thinking compared to older children (ages 8–12), with the tool capturing these differences appropriately.
*Hypothesis 3:* It is hypothesized that the Italian validation of the CTAC will show psychometric characteristics consistent with those reported in similar international studies on children’s critical thinking assessment tools, thereby supporting its cross-cultural applicability for future research.

Finally, while this study focuses on the validation and standardization of the CTAC within the Italian context, future cross-cultural research will be needed to directly compare its psychometric performance with that of other critical thinking assessments and across different populations.

## Overall Method

### Preliminary Phase of CTAC Construction

The construction of the Critical Thinking Assessment for Children (CTAC) followed a systematic process, including item generation, expert evaluation, pilot testing for discrimination index analysis, and item refinement. This phase established a robust psychometric foundation for subsequent validation procedures. A comprehensive literature review was conducted to identify existing scales and items related to critical thinking in children. The review was drawn from validated instruments and well-established theoretical frameworks (Fabio et al., 2023; Fabio & Suriano, 2023; Facione & Facione, 2013). Based on this literature, a preliminary list of 24 items was generated to assess various dimensions of critical thinking in children. These dimensions included the ability to identify contradictions, integrate life experiences into critical thinking, formulate questions, evaluate the truthfulness of advertisements, propose alternative solutions to problems, recognize conflicting statements, detect biases, and interpret humor. Table 1 presents the complete list of item categories and their sources.

**Table 1:** Categories of Critical Thinking Items and Corresponding Sources

Category	Sources
• Contradictions identification	Delamain and Spring (2021); Dobrusskin (2016); Ma and Ganea (2010)
• Life experience integration for Critical Thinking	Butler et al. (2012); Tunjungsari and Takwin (2021); Delamain and Spring (2021); Franco et al., (2017).
• Questioning Proficiency	Aras and Aslan (2018); Bilir Seyhan et al. (2019); Cesur and Yaralı (2020); Delamain and Spring (2021); Kirkland et al. (2015); Nikiforidou (2017); Walan and Enochsson (2019); Rezaei et al. (2011)
• Analyzing Truth in Advertisments	Brosseau-Liard (2017); Delamain and Spring (2021); Halpern, 2013; Hübscher et al. (2017)
• Problem-solving alternatives	Butera et al. (2014); Delamain and Spring (2021); Bridgers et al. (2016)
Detection of conflicting statements	Bridgers et al. 2016; Delamain and Spring (2021)
Bias recognition	Atwood (2022); Delamain and Spring (2021)
Joke decoding ability	Delamain and Spring (2021); Tian et al. (2017)

The items, along with relevant visual materials, are detailed in Appendix A of Fabio and Patafi (2026). For this article, data, codebook and materials are available at Fabio (2025). To ensure content validity, an independent panel of eight experts in the field of child cognitive development and critical thinking assessment evaluated the items. The panel included researchers and practitioners with extensive experience in psychometric test development, educational assessment, and child psychology. Experts were selected based on their publication record and prior involvement in critical thinking research or educational measurement projects.

Each expert independently rated the items using a structured evaluation form that assessed multiple aspects:

- Relevance — how well each item captures the intended critical thinking dimension.
- Representativeness — whether any items fail to adequately represent critical thinking.
- Clarity — clarity of instructions, wording, and visual materials.
- Potential Misinterpretation — identification of items likely to confuse children or elicit unintended interpretations.
- Comprehensiveness — whether the set of items collectively covers the targeted dimensions of critical thinking.
- Missing Dimensions — any aspects of critical thinking or specific behaviors not represented.

Experts provided both quantitative ratings (on a 5-point Likert scale) and qualitative feedback, including suggested revisions to wording or content. Inter-rater agreement was assessed, and items showing low consensus were discussed in a follow-up panel meeting to reach consensus. The evaluations indicated strong agreement across the panel regarding the high relevance, clarity, and comprehensiveness of the items. Minor modifications were made to improve wording and reduce potential ambiguity, ensuring that the final item pool accurately reflected the construct of critical thinking in children.

Subsequently, a pilot test was conducted with 72 children aged 6 to 8 to identify potential issues with the items and assess their discriminative ability. The Discrimination Index (D) was applied (Chiorri, 2011) to measure each item’s effectiveness in distinguishing between high- and low-performing participants. Items with low discrimination indices indicate that responses were similar across participants, regardless of their total test score. The results from the pilot test demonstrated that most items exhibited strong discriminative capabilities. Items with a discrimination index below 0.20 were excluded, resulting in the final retention of 22 items. Specifically, two items were removed: one related to Life Experience Integration for Critical Thinking and another concerning Bias Recognition. The low discrimination indices for these items suggest that they posed significant challenges for all children in the sample, potentially indicating issues with wording or conceptual difficulty.

## Study 1

### Method

The literature analysis led to the development of the final version of the Critical Thinking Assessment for Children (CTAC), which was administered to 18 children (9 females; mean age: 7.43 ± 1.12) to further refine the assessment's structure.

#### Participants

The study was conducted in two primary schools in Italy. A total of 112 children (60 girls) were recruited from second to fifth grade. Inclusion criteria required children to be typically developing, attending the regular primary school curriculum, and fluent in Italian. Children with diagnosed neurological, psychiatric, or developmental disorders were excluded. These children participated voluntarily, with parental consent. The sample consisted of one class from each grade level: 39 second-grade children (Age range 6.1–7.9 years; *M* = 7.43 ± 1.2), 31 third-grade children (Age range 8.1–9.11 years; *M* = 8.45 ± 1.2), and 42 fourth- and fifth-grade children (Age range 10–11.10 years; *M* = 10.51 ± 1.3),). All participating children were Italian and came from mixed socioeconomic backgrounds. Informed consent was obtained from the parents of the participants. The study received approval from the University Human Research Ethics Committee (Protocol 003611112, 13 January 2024).

#### Procedure

The examiner and child sat face-to-face in a quiet, familiar, and adequately lit classroom environment. The examiner read a brief introductory text to the child, then asked three questions. The child was given a paper booklet containing all the items, with explanatory pictures where necessary to aid understanding. To ensure standardization, voice recordings of the examiner reading the questions were played back to the child. The administration of the instrument took approximately 15 minutes per child.

The questions were based on everyday situations and contingencies that children were likely to have encountered during their development. These questions encouraged children to question the truth and motives behind statements (Brosseau-Liard, 2017). The answers were scored quantitatively, with one point awarded for each correct answer, up to a maximum of 3 points per item. In the original 24-item version, the maximum total score was 72; after item reduction, the maximum total score was 66 in Study 1 (22 items) and 48 in Study 2 (16 items). The items, along with the detailed scoring rubric for the original 24-item version, are provided in Appendix A in Fabio and Patafi (2026).

##### Example: Truth in Advertising

(see in Appendix A, Fabio & Patafi, 2026)

“The chocolate with the red label is the best in the world!”

- In your opinion, why is it stated that chocolate with the red label is the best in the world?
- Who might have written this?
- Do you think it’s true that it is the best? How could one prove it?

**Correct Answers:**

1. "They wish to sell it; they made an advertisement" (1 point).
2. "The owner of the chocolate; the seller" (1 point).
3. "I don’t think it’s the best because I haven’t tasted it; maybe, I’m not sure because I can’t prove it" (1 point).

**Incorrect Answers:**

“I don’t know; It’s the best one; It’s the best because it’s made with milk” (0 points).
“I don’t know; The mother; Children who like chocolate” (0 points).

#### Flow of Participants and Item Development

The development of the Critical Thinking Assessment for Children (CTAC) followed a multi-stage process to ensure both content validity and psychometric robustness. The following section outlines the item refinement pipeline from the initial 24 items to the final 16-item version validated in Study 2.

In the preliminary study, items were reduced from 24 to 22 based on discrimination indices, removing two items that showed low differentiation across participants. This 22-item version retained the original scoring of 3 points per item, yielding a maximum total score of 66.

In Study 1, the 22-item version was administered to a larger sample, and exploratory factor analysis revealed three factors: Open-Minded Inquiry, Media Literacy and Critical Analysis, and Contradiction Recognition and Critical Awareness.

In Study 2, the 22-item version was further validated in a larger sample (*N* = 356). Factor loadings and item performance guided the creation of a shortened 16-item version, optimized for brevity and age-appropriateness while maintaining psychometric quality. Scoring remained consistent at 3 points per item, for a maximum total of 48 points.

Appendix A (see Fabio & Patafi, 2026) reports the original 24-item Italian version of the CTAC, used in the preliminary study, along with the scoring rubric for assigning points to responses. Following item analysis, two items were removed due to low discrimination, yielding the 22-item Italian version used in Study 1 (Appendix B, Fabio & Patafi, 2026). Finally, based on factor loadings and item performance in Study 2, the shortened 16-item version was created, presented in Appendix C (see Fabio & Patafi, 2026) in both Italian and English, together with the rubric specifying scoring criteria for each item. This progression (24 → 22 → 16 items) ensures transparency, traceability, and retention of content validity across studies.

### Results

The Kaiser-Meyer-Olkin (KMO) measure of sampling adequacy was 0.91, indicating that the correlation matrix was suitable for factor analysis. Principal axis factoring with Direct Oblimin oblique rotation was employed. Three criteria were used to extract factors: parallel analysis of Monte Carlo simulations, eigenvalues greater than 1 (Kaiser’s criterion), and visual inspection of the scree plot for significant contributions to the explained variance. The factor analysis identified a three-factor solution. Table 2 displays the factor loadings for all items, showing high loadings on three factors. The first factor accounted for 30% of the variance, the second for 10%, and the third for 10%, resulting in a cumulative explained variance of 50%.

**Table 2:** Factor Loadings

Item	Factor 1: Open-Minded Inquiry (9 items)	Factor 2: Media Literacy and Critical Analysis (3 items)	Factor 3: Contradiction Recognition and Critical Awareness (4 items)
Questioning Proficiency 1	0.651		
Problem-Solving Alternatives 1	0.585		
Problem-Solving Alternatives 2	0.562		
Questioning Proficiency 2	0.511		
Questioning Proficiency 3	0.505		
Life Experience Integration for Critical Thinking 2	0.502		
Problem-Solving Alternatives 3	0.470		
Detection of Conflicting Statements 1	0.449		
Detection of Conflicting Statements 2	0.424		
Analyzing Truth in Advertisements 2		0.940	
Bias Recognition 1		0.623	
Analyzing Truth in Advertisements 3		0.625	
Contradiction Identification 3			0.670
Contradiction Identification 2			0.661
Detection of Conflicting Statements 3			0.686
Contradiction Identification 1			0.629

*Note.* Table 2 presents the final 16 items retained after the exploratory factor analysis (Study 1). Joke Decoding items and Bias Recognition items that did not meet the .40 loading criterion were excluded prior to confirmatory testing; however, Bias Recognition 1 was retained because it loaded on Factor 2. Factor 1 corresponds to Open-Minded Inquiry (9 items), Factor 2 to Media Literacy and Critical Analysis (3 items), and Factor 3 to Contradiction Recognition and Critical Awareness (4 items). The specific wording of all 16 retained items is provided in Appendix C (see Fabio & Patafi, 2026), in both the original Italian version and the English translation. This 9/3/4 configuration was carried forward and confirmed in Study 2 (CFA).

Nine items loaded strongly on the first factor, labeled *Open-Minded Inquiry*, which represents the child’s tendency to be receptive to various perspectives and engage in critical thinking. This factor comprises three subcomponents: asking questions, suggesting alternatives, and evaluating conflicting statements (Bridgers et al., 2016). The second factor, labeled *Media Literacy and Critical Analysis*, reflects the ability to recognize that advertising messages are not absolute truths and to critically analyze media content. The third factor, *Contradiction Recognition and Critical Awareness*, emphasizes the ability to identify contradictions and fosters critical awareness in interpreting information. The Cronbach’s *alphas* for the three factors were .83, .73, and .81, respectively, and .93 for the full scale. All subscales showed significant correlations with the CTAC and with each other (see Table 3).

**Table 3:** Descriptive Statistics and Correlations of CTAC and Subscales

Variable	*M*	*SD*	1	2	3	4
1. Open minded inquiry	17.83	5.63	—			
2. Media literature analysis	4.48	2.66	.530**	—		
3. Contradiction recognition	8.67	3.04	.497**	.445**	—	
4. TOT Critical Thinking	30.98	9.39	.911**	.745**	.748**	—

The solution excluded the items of the Joke Decoding Ability subscale and the Bias Recognition items that did not exhibit significant factor loadings on any of the primary factors identified. Bias Recognition 1 was retained because it loaded significantly on Factor 2.

A full trace of the item development and reduction process is provided in Appendix C (see Fabio & Patafi, 2026), which lists all items included in the final 16-item CTAC together with their factor loadings and subscale assignments. The final structure comprised nine items for Open-Minded Inquiry, three for Media Literacy and Critical Analysis, and four for Contradiction Recognition and Critical Awareness. The removed items (Bias Recognition and Joke Decoding) did not load significantly on any factor and were therefore excluded prior to confirmatory validation.

## Study 2

### Method

#### Participants

The study was conducted in a primary school in Italy. A total of 356 children (181 girls) were recruited from second- to fifth-grade classes. Participation was voluntary, with parental consent obtained. The sample included 108 second-grade children (age range = 6.1–7.9 years, *M* = 7.43, *SD* = 1.20), 128 third-grade children (age range = 8.1–9.11 years, *M* = 8.45, *SD* = 1.20), and 120 fourth- and fifth-grade children (age range = 10–11.10 years, *M* = 10.51, *SD* = ± 1.3). The sample was predominantly Italian (*n* = 340), with a few international participants: two Cuban children, eight from Morocco, four from Libya, and two from Albania. All children spoke Italian fluently, with the exception of one Cuban girl. All participants were well integrated into the classroom context.

#### Measures

The CTAC was used to assess critical thinking behaviors. The final 16-item version from Study 1 was administered, measuring three factors: Open-Minded Inquiry (9 items), Media Literacy and Critical Analysis (3 items), and Contradiction Recognition and Critical Awareness (4 items). Responses were scored quantitatively, with a maximum total score of 48. The internal consistency was high (α = .93).

Additionally, the Raven’s Coloured Progressive Matrices (CPM; Raven, 1940) were administered to 149 randomly selected children from the total sample to assess fluid intelligence. The internal consistency for the CPM was also high (α = .87).

Teacher-assigned grades in linguistic, mathematical, and artistic disciplines were collected to assess concurrent validity.

#### Statistical Analyses

Confirmatory factor analysis (CFA) was performed using JASP open-source software to determine the best latent structure for the CTAC. Various fit indices were examined, including χ^2^/*df*, Goodness of Fit Index (GFI), Comparative Fit Index (CFI), and Root Mean Square Error of Approximation (RMSEA).

Second-order CFA confirmed that the CTAC dimensions represented the overarching construct of critical thinking. Correlations with other constructs were assessed using Pearson’s correlation coefficients for the CTAC and CPM scores, and Spearman’s rank correlation coefficients for teacher-assigned grades. Internal consistency was evaluated using Cronbach’s alpha.

### Results

#### Confirmatory Factor Analysis

**Figure 1:**
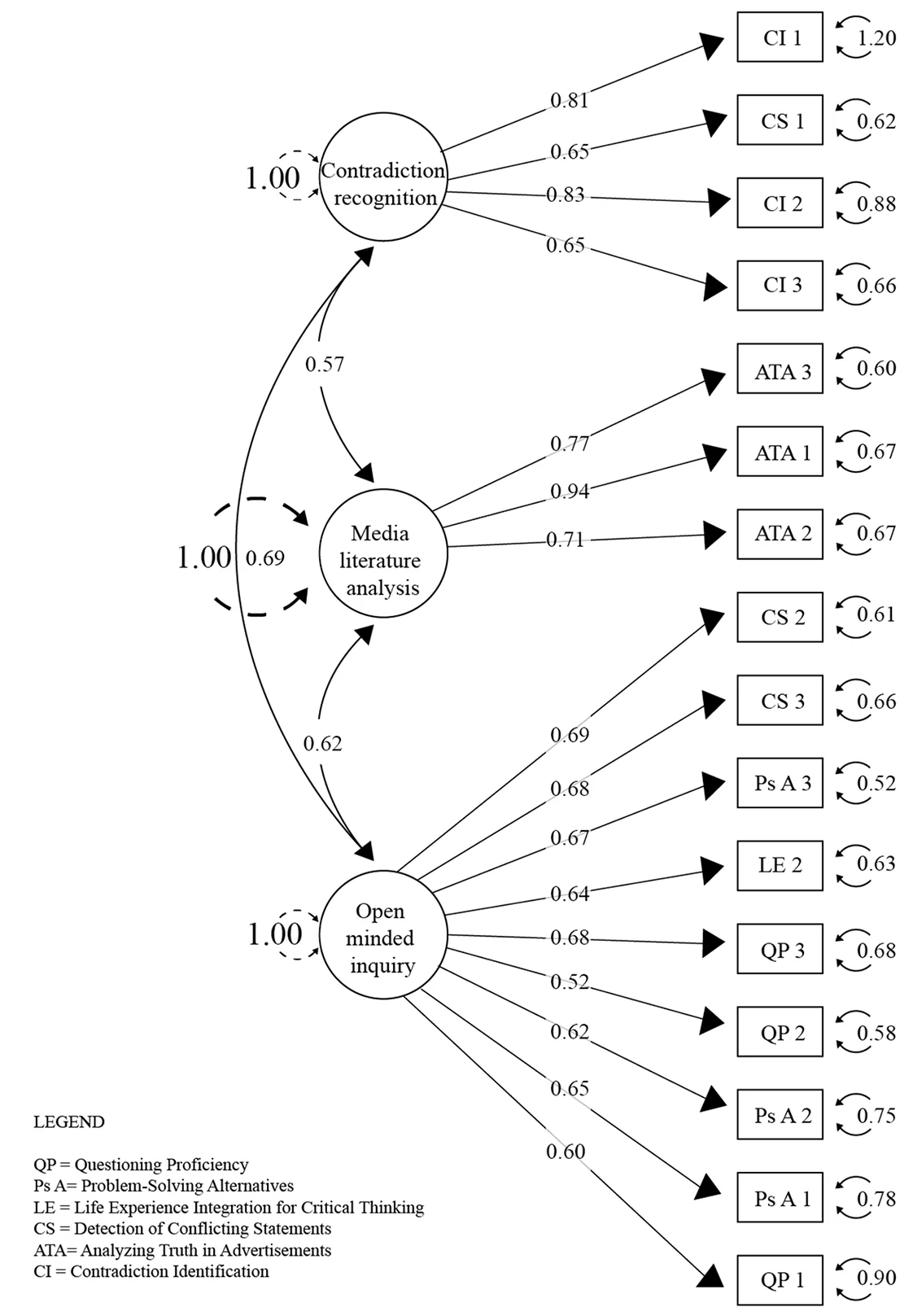
Second-Order Three-Factor Model of the CTAC Confirming the Overarching Structure OF Critical Thinking With Standardized Factor Loadings Shown

A confirmatory factor analysis (CFA) was conducted on the 16-item CTAC using JASP to test the proposed three-factor structure derived from Study 1. Two models were compared: a basic three-factor model and a second-order model representing Critical Thinking as a higher-order latent construct. A chi-square difference test indicated that the second-order model fit the data significantly better than the basic three-factor model, *Δ*χ^2^*(*4) = 70.65, *p* < .001. Accordingly, the second-order model was retained as the final structure (Figure 1). Initially, the model exhibited acceptable but suboptimal fit indices, χ^2^*(*105) = 122.46, *p* > .05, *CFI* = .91, *RMSEA* = .082 (90% *CI* [.069, .092]). Inspection of modification indices suggested that four residual covariances could be freely estimated between items within the same subscale. These items shared similar wording or conceptual content (e.g., reflective evaluation or media judgment), justifying their covariance on theoretical grounds rather than as data-driven adjustments. After introducing these theoretically consistent modifications, the model fit improved substantially, χ^2^(101) = 51.81, *p* > .05, *CFI* = .96, *RMSEA* = .068 (90% *CI* [.049, .076]).

All standardized factor loadings were ≥ .60, indicating strong associations between items and their respective latent factors. A second-order factor representing overall critical thinking also showed significant loadings on each first-order dimension, confirming that Open-Minded Inquiry, Media Literacy and Critical Analysis, and Contradiction Recognition and Critical Awareness together constitute a coherent overarching construct.

Internal consistency was excellent across the CTAC dimensions. Cronbach’s α and McDonald’s ω were, respectively, .93 and .92 for the total scale, .91/.90 for Open-Minded Inquiry, .88/.86 for Media Literacy and Critical Analysis, and .85/.83 for Contradiction Recognition and Critical Awareness, indicating high reliability and structural coherence.

#### Reliability

Cronbach’s alpha for the 16-item CTAC was .93, indicating satisfactory to excellent internal consistency.

#### Convergent and Criterion Validity

To assess convergent validity, Pearson’s correlation coefficients were calculated between CTAC scores and Raven’s CPM scores, as well as linguistic performance scores. As shown in Table 4, CTAC scores were significantly and positively correlated with both Raven’s CPM and linguistic grades. These results suggest that higher critical thinking scores are associated with greater fluid intelligence and better linguistic performance.

**Table 4:** Correlations Between CTAC, Raven Scoring, Linguistic Scoring, Math Scoring and Art Scoring

Variable	Raven scoring	Linguistic scoring	Math scoring	Art scoring
1. CTAC scale	.48**	.41**	.11	.09
2. Open-Minded Inquiry	.49**	.29**	.12	.03
3. Media Literacy and Critical Contradiction recognition	.36**	.30**	.21	.11
4. Contradiction Recognition and Critical Awareness	.33**	.42**	.13	.08

No significant correlation was found between CTAC scores and mathematical grades, suggesting that critical thinking skills may align more closely with linguistic than with mathematical proficiency. Similarly, no significant correlation emerged between CTAC scores and artistic performance grades.

#### Age and Critical Thinking

The analysis of age as a factor revealed its significance across the entire CTAC scale, as well as within each individual subscale (*F*(2, 353) = 36.45, *p* < .001, η^2^ = .09). Consequently, age was found to influence critical thinking performance, and standardization indices were provided for distinct age groups. Table 5 presents the means and standard deviations for each homogeneous age group, which included three statistically homogeneous cohorts: 6.1–7.9 years, 8.1–9.11 years, and 10.00–11.10 years. This approach improves the interpretability of the CTAC scores by accounting for age-related differences and facilitating cross-age comparisons.

**Table 5:** Means and Standard Deviations for Each Age Group

Age Group	6.1–7.9 years	8.1–9.11 years	10.00–11.1years
CTAC Total Score	25.83 (±8.55)	30.35 (±8.70)	36.36 (±6.64)
Open-Minded Inquiry	15.47 (±4.56)	16.89 (±5.11)	20.97 (±4.11)
Media Literacy and Critical Analysis	3.06 (±1.21)	4.63 (±2.06)	5.59 (±2.12)
Contradiction Recognition and Critical Awareness	7.30 (±2.11)	8.81 (±3.11)	9.79 (±2.86)

## Overall Discussion

This study aimed to address a critical gap in the literature by developing and validating the Critical Thinking Assessment for Children (CTAC), a tool specifically designed to assess critical thinking in Italian children. The findings offer several important insights that both align with and extend the existing body of research on critical thinking development.

Drawing on developmental models proposed by Piaget (1962) and expanded by Daniel and Gagnon (2012), this study conceptualized critical thinking as evolving through successive cognitive stages that become more sophisticated with maturation. The second-order three-factor model identified through the CTAC — Open-Minded Inquiry, Media Literacy and Critical Analysis, and Contradiction Recognition and Critical Awareness — reflects these developmental stages. This nuanced framework enhances our understanding of how critical thinking manifests during childhood and aligns with previous research emphasizing the multidimensional nature of critical thinking (Facione & Facione, 2013; Ku, 2009).

The Confirmatory Factor Analysis (CFA) provided strong support for the robustness of this model. All items exhibited substantial factor loadings (all above .60), indicating that they are well-aligned with their respective constructs and confirming that the CTAC accurately captures the theoretical dimensions of children's critical thinking. This finding contributes valuable empirical evidence to a relatively underexplored area, offering a structurally valid measure tailored specifically for young children (Crisafulli et al., 2017).

### Correlational Findings and Their Implications

The positive correlations between the CTAC and both Raven’s Coloured Progressive Matrices and measures of linguistic abilities provide additional support for its validity. These findings suggest that the CTAC captures cognitive reasoning processes closely related to general intelligence and language skills. This is consistent with previous research linking critical thinking to broader cognitive abilities (Halpern, 2013; Stanovich, 2011) and highlights the potential of the CTAC as a predictor of academic performance in domains requiring logical reasoning and language proficiency.

Interestingly, the absence of a significant correlation between CTAC scores and mathematical performance suggests that the critical thinking skills assessed may be more verbal and contextual rather than numerical or purely analytical. This result merits further investigation, especially in light of research indicating that critical thinking encompasses both verbal and analytical reasoning (Kahneman, 2011; Stanovich, 2011). Future adaptations of the CTAC might consider incorporating more numerically oriented tasks to ensure a more comprehensive assessment of children's critical thinking abilities.

### Integration With Existing Literature

The CTAC builds upon and addresses limitations observed in previous critical thinking assessments. Multiple-choice formats, while widely used, often fail to capture the complexity of critical thinking processes (Ku, 2009). Although performance-based tasks are more comprehensive, they can be resource-intensive and heavily context-dependent (Shavelson et al., 2019). The CTAC strikes a balance by using verbal and contextual tasks that are both practical and theoretically grounded, aligning with recommendations for feasible and valid critical thinking assessments in educational settings (Facione, 2015).

Furthermore, the inclusion of media literacy within the CTAC structure reflects an important, contemporary dimension of critical thinking development. In an age of information overload, discerning credible sources has become a crucial skill (Halpern, 2013). By incorporating media literacy, the CTAC not only assesses traditional critical thinking competencies but also addresses modern challenges, enhancing its relevance for today’s digital environment.

### Limitations and Future Directions

Despite its promising findings, this study has several limitations. The sample was drawn from two specific regions in Italy, which may restrict the generalizability of the results. Future research should aim to validate the CTAC in more diverse populations, both nationally and internationally, to establish its broader applicability.

Moreover, the cross-sectional design offers only a static snapshot of critical thinking development. Longitudinal studies are needed to provide dynamic insights into how critical thinking skills evolve over time and the various factors that influence this process. Another avenue for future research is the refinement of the CTAC to include a wider variety of tasks, particularly those targeting numerical reasoning, to capture the full breadth of critical thinking competencies. Further validation efforts could also examine the predictive validity of the CTAC concerning long-term academic and life outcomes.

### Conclusion

In summary, the CTAC represents a significant advancement in the assessment of critical thinking in children. It offers a reliable, valid, and theoretically grounded tool that addresses many limitations of existing measures. By integrating established developmental theories and contemporary challenges such as media literacy, the CTAC enhances our understanding of critical thinking development in childhood. It also provides practical insights for educators and policymakers aiming to cultivate these essential skills in future generations. Continued research and refinement will further strengthen the CTAC’s utility and contribute meaningfully to the growing body of knowledge on critical thinking within educational contexts.

## Supplementary Materials

**Table:** 

Type of supplementary material	Availability/Access
Data	
Data set critical thinking	Fabio (2025)
Code	
SPSS_Syntax_CTAC	Fabio (2025)
Material	
English instrument CTAC table	Fabio (2025)
Italian original instrument for CTAC	Fabio (2025)
Full tables with literature generation of each item	Fabio (2025)
Study/Analysis preregistration	
Study was not preregistered	—
Other	
Codebook_CTAC	Fabio (2025)
Appendices with three versions of Critical Thinking Assessment for Children: items and visual materials, and scoring rubric	Fabio and Patafi (2026)

## Data Availability

For this article, data, codebook and materials are available at Fabio (2025).
